# Pyridoxine-Deficient Streptococcus: An Uncommon Virulent Cause of Subacute Destructive Aortic Valve Endocarditis

**DOI:** 10.7759/cureus.22951

**Published:** 2022-03-08

**Authors:** Abdullah Ahmad, Zachary Banbury, Enrique Baez, Smitha Agadi, Aalap Chokshi

**Affiliations:** 1 Internal Medicine, Englewood Hospital and Medical Center, Englewood, USA; 2 Cardiology, Englewood Hospital and Medical Center, Englewood, USA

**Keywords:** cardiovascular system, abiotrophia, pyridoxal-dependent streptococcus, nutritional variant streptococcus, infective endocarditis

## Abstract

Nutritional variant streptococcus (NVS) or pyridoxal-dependent streptococcus is a rare but significant cause of infective endocarditis (IE), which presents as a diagnostic dilemma due to difficulty in organism isolation, and high rates of treatment failure, recurrence, and mortality. We discuss a case of a 52-year-old male who presented with chronic fatigue, cyclic fever, night sweats, and weight loss. He was treated with culture-directed antibiotics and surgical aortic valve replacement due to disease severity and risk of embolization. This case highlights the clinical significance of NVS IE, and the importance of early recognition, and immediate, often invasive therapy to improve outcomes.

## Introduction

Nutritional variant streptococcus (NVS) is an uncommon but serious cause of 6% of streptococcus-related infective endocarditis (IE) and 1-2% of all cases of IE. It is associated with a high risk of complications and mortality. NVS usually resides in the oral cavity, intestinal tract, or genitourinary tract, and can manifest as culture-negative endocarditis, osteomyelitis, or septic arthritis with a high propensity for septic emboli (30%) [1-5]. Here we present an unusual case of NVS subacute endocarditis of the aortic valve (AV) complicated by severe aortic regurgitation (AR) necessitating aortic valve replacement (AVR).

## Case presentation

A 52-year-old male with a history of hypertension, chronic back pain, iron, and deficiency anemia, with a negative workup for gastrointestinal source of bleeding presented to the emergency department complaining of two months of fatigue, cyclic fever, night sweats, unintentional weight loss, and dry cough. The patient denied paresthesia in the lower extremities, dyspnea, chest pain, abdominal pain, diarrhea, or rash. His physical examination showed normal vital signs including blood pressure of 126/47 mmHg, pulse rate of 94 beats per minute, and respiratory rate of 18 breaths per minute, except for a temperature of 38.5 degrees Celsius, generally ill-appearance, thin, pallor, right upper sternal border grade II/VI diastolic murmur, clear lungs, absence of rash, and lymphadenopathy. Outpatient computed tomography (CT) scan excluded any thoracic/abdominal malignancy.

**Figure 1:**
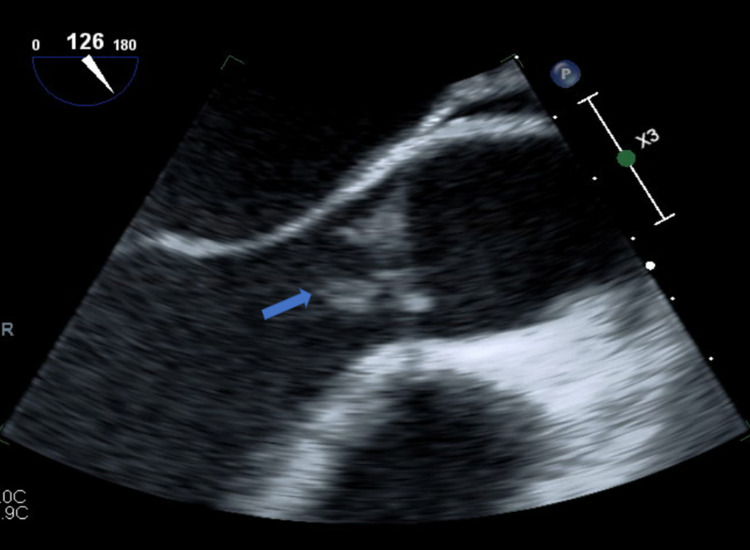
TEE showing two separate mobile vegetations (blue arrow). TEE, transesophageal echocardiography

**Figure 2:**
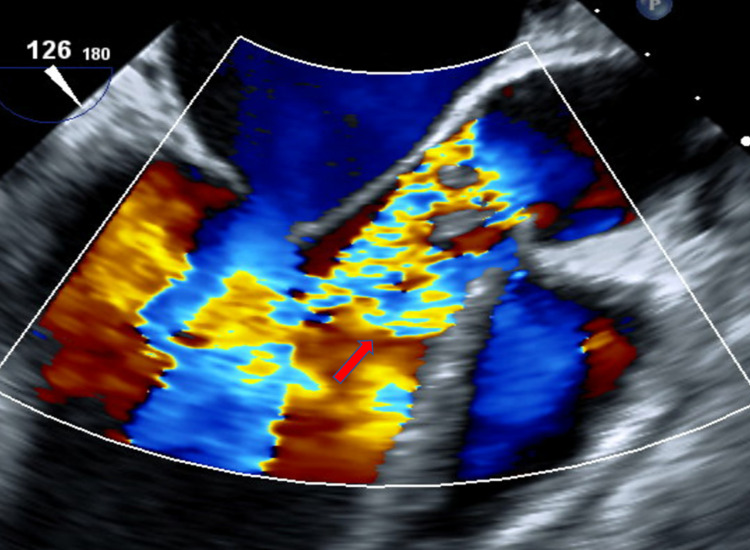
TEE showing severe aortic valve regurgitation (red arrow). TEE, transesophageal echocardiography

Basic laboratory evaluation is given in Table 1. Electrocardiogram showed sinus tachycardia. Chest X-ray was normal. Two blood culture bottles grew nutritionally (B6) deficient streptococcus on chocolate agar after no growth on blood agar. Vancomycin with cefepime empiric antibiotics coverage was started, which was tailored as per culture sensitivity to vancomycin only. Transthoracic echocardiogram (TTE) was remarkable for 0.12 x 0.17 cm vegetation on the coronary cusp of AV, mild-moderate AR, small pericardial effusion, and preserved left ventricular ejection fraction ([EF] 50-55%). Transesophageal echocardiography (TEE) showed two separate mobile vegetations (Figure 1) protruding into the left ventricular cavity (that on the non-coronary cusp measured 0.8 x 1.0 cm and the other on the right coronary cusp measured 0.9 x 0.6 cm) and severe AR (Figure 2 ). Diagnosis of IE was made in keeping with Dukes criteria with the presence of two major criteria (two blood cultures positive for NVS and evidence of endocardial involvement on TTE) and one minor criterion (fever). Lumbar spine magnetic resonance imaging (MRI) showed L5-S1 discitis/osteomyelitis.

**Table 1:** Laboratory workup Abbreviations: Lab, Laboratory; BNP, brain natriuretic peptide; CRP, C-reactive protein; ESR, erythrocyte sedimentation rate; RF, rheumatoid factor; ALP, alkaline phosphatase; ALT, alanine transaminase; AST, aspartate aminotransferase; Hb, hemoglobin; WBC, white blood cell; TSH, thyroid-stimulating hormone; T4, thyroxine; IgG, immunoglobulin G; IgM, immunoglobulin M; HIV, human immunodeficiency virus; TSAT, transferrin saturation

Lab	Result	reference
BNP	101	0–100 pg/mL
Troponin I	0.05	0.00–0.03 ng/mL
CRP	139.9	< 10 mg/L
ESR	65	0–20 Mm/Hr
RF	35	< 12 IU/mL
Liver enzymes		
ALP	154	38–126 U/L
ALT	81	< 50 U/L
AST	97	17–59 U/L
Hb	7.3	13.0–18.0 g/dL
WBC	8.45	4.00–11.00 K/uL
Thyroid function test		
TSH	4.470	0.270–4.200 UIU/mL
T4	1.0	0.8–2.2 ng/dL
Lactic acid	1.2	0.7–2.0 mmol/L
Lyme		
IgG	Negative	Negative
IgM	Negative	Negative
HIV 1&2		
Antibody screen	Nonreactive	Nonreactive
Antigen screen	Nonreactive	Nonreactive
Blood parasites smear	No parasite seen	No parasite seen
Iron panel		
Iron level	31	49–181 ug/dL
TSAT	12	15–50 %

Although the patient remained clinically asymptomatic from a cardiac standpoint, given the size of the vegetations, possible hemodynamic implications, and the risk of septic emboli, the patient was planned for surgical AVR. Prior to surgery, hemoglobin trended up with iron infusions, seven weeks of intravenous ceftriaxone were completed, and pre-operative coronary angiogram excluded coronary artery disease. Intraoperative culture of AV tissue showed no growth. Post-surgical AVR, the patient developed new-onset cardiomyopathy with EF of 20-25%. He was placed on guideline-directed medical therapy and prescribed a wearable cardioverter defibrillator. Repeat TTE at three months showed improvement in EF to 45-50%, eliminating the need for primary prevention defibrillator. Etiology of cardiomyopathy was likely due to postsurgical myocardial stunning.

The patient completed a three-month course of anti-coagulation with rivaroxaban and aspirin and is now maintained on aspirin alone. He has regained his pre-morbid weight and has significant improvement in inflammatory markers and symptomatology.

## Discussion

Abiotrophia, also classified as NVS, are catalase-negative, oxidase-negative, facultative anaerobic, Gram-positive cocci. They usually affect immunocompromised individuals as well as those with prosthetic valves [5]; however, our patient was previously well with no intravenous drug use or other characteristic underlying risk factors. NVS is usually difficult to isolate via culture due to their pleomorphic nature, specific culture media characteristics, nutritional requirements, and slow growth rate. Fortunately, for our patient, this virulent organism was quickly identified and successfully treated despite a treatment failure rate of 41%, high recurrence rate of 17%, and mortality of more than 17%. This is often attributed to delays in identification and high prevalence of beta-lactams and macrolide antibiotic resistance despite the efficacy of antibiotics in vitro [3-7]. The high virulence of NVS is attributed to exopolysaccharide production and the affinity to fibronectin [8]. NVS is very fastidious and will grow only on supplemented media or enriched chocolate agar [9]. In persistent blood culture-negative cases with high clinical suspicion and/or imaging supporting the diagnosis, 16S ribosomal RNA (rRNA) gene sequencing technology or matrix-assisted laser desorption ionization time of flight mass spectrometry (MALDI-TOF-MS) can be used to identify the organism [3-7,10].

NVS has a propensity to heart valves, and the resultant endocarditis characteristically has a poor prognosis. The AV is more commonly affected than other valves, consistent with our case, and AVR is indicated in 30% of cases [5]. The American Heart Association recommends treatment with penicillin or ceftriaxone and gentamycin for four to six weeks (vancomycin in penicillin-allergic patients) [1-6]. Early surgery is associated with better outcomes in patients with severe valvular disease and large vegetations [4].

## Conclusions

NVS is a rare organism that can often lead to clinically significant endocarditis. In an initial negative culture, consider using supplemented media or enriched chocolate agar. It is unusual in a healthy patient without predisposing risk factors such as intravenous drug use, prosthetic heart valve, immunocompromised state, and clear source of entry. However, early recognition and immediate, often invasive, therapy is curative.
